# Limitations and potential strategies of immune checkpoint blockade in age-related neurodegenerative disorders

**DOI:** 10.1186/s12576-024-00933-4

**Published:** 2024-09-23

**Authors:** Noha N. Lasheen, Salma Allam, Abdullrahman Elgarawany, Darin W. Aswa, Rana Mansour, Ziad Farouk

**Affiliations:** 1 Department of Basic Medical Sciences, Faculty of Medicine, Galala University, Suez, Egypt; 2 Department of Physiology, Faculty of Medicine, Ain Shams University, Cairo, Egypt; 3 Faculty of Medicine, Galala University, Galala City, Suez Egypt

**Keywords:** Immune checkpoint, Age-related neurodegenerative disorders, Alzheimer's disease, Parkinson's disease, Monoclonal antibodies

## Abstract

Neurological disorders such as Alzheimer’s disease (AD), and Parkinson’s disease (PD) have no disease-modifying treatments, resulting in a global dementia crisis that affects more than 50 million people. Amyloid-beta (Aβ), tau, and alpha-synuclein (α-Syn) are three crucial proteins that are involved in the pathogenesis of these age-related neurodegenerative diseases. Only a few approved AD medications have been used in the clinic up to this point, and their results are only partial symptomatic alleviation for AD patients and cannot stop the progression of AD. Immunotherapies have attracted considerable interest as they target certain protein strains and conformations as well as promote clearance. Immunotherapies also have the potential to be neuroprotective: as they limit synaptic damage and spread of neuroinflammation by neutralizing extracellular protein aggregates. Lately, disease-modifying therapies (DMTs) that can alter the pathophysiology that underlies AD with anti-Aβ monoclonal antibodies (MAbs) (e.g., aducanumab, lecanemab, gantenerumab, donanemab, solanezumab, crenezumab, tilavonemab). Similarly, in Parkinson's disease (PD), DMTs utilizing anti-αSyn (MAbs) (e.g., prasinezumab, cinpanemab,) are progressively being developed and evaluated in clinical trials. These therapies are based on the hypothesis that both AD and PD may involve systemic impairments in cell-dependent clearance mechanisms of amyloid-beta (Aβ) and alpha-synuclein (αSyn), respectively, meaning the body's overall inability to effectively remove Aβ and αSyn due to malfunctioning cellular mechanisms. In this review we will provide possible evidence behind the use of immunotherapy with MAbs in AD and PD and highlight the recent clinical development landscape of anti-Aβ (MAbs) and anti-αSyn (MAbs) from these clinical trials in order to better investigate the therapeutic possibilities and adverse effects of these anti-Aβ and anti-αSyn MAbs on AD and PD.

## Introduction

**Fig. 1:**
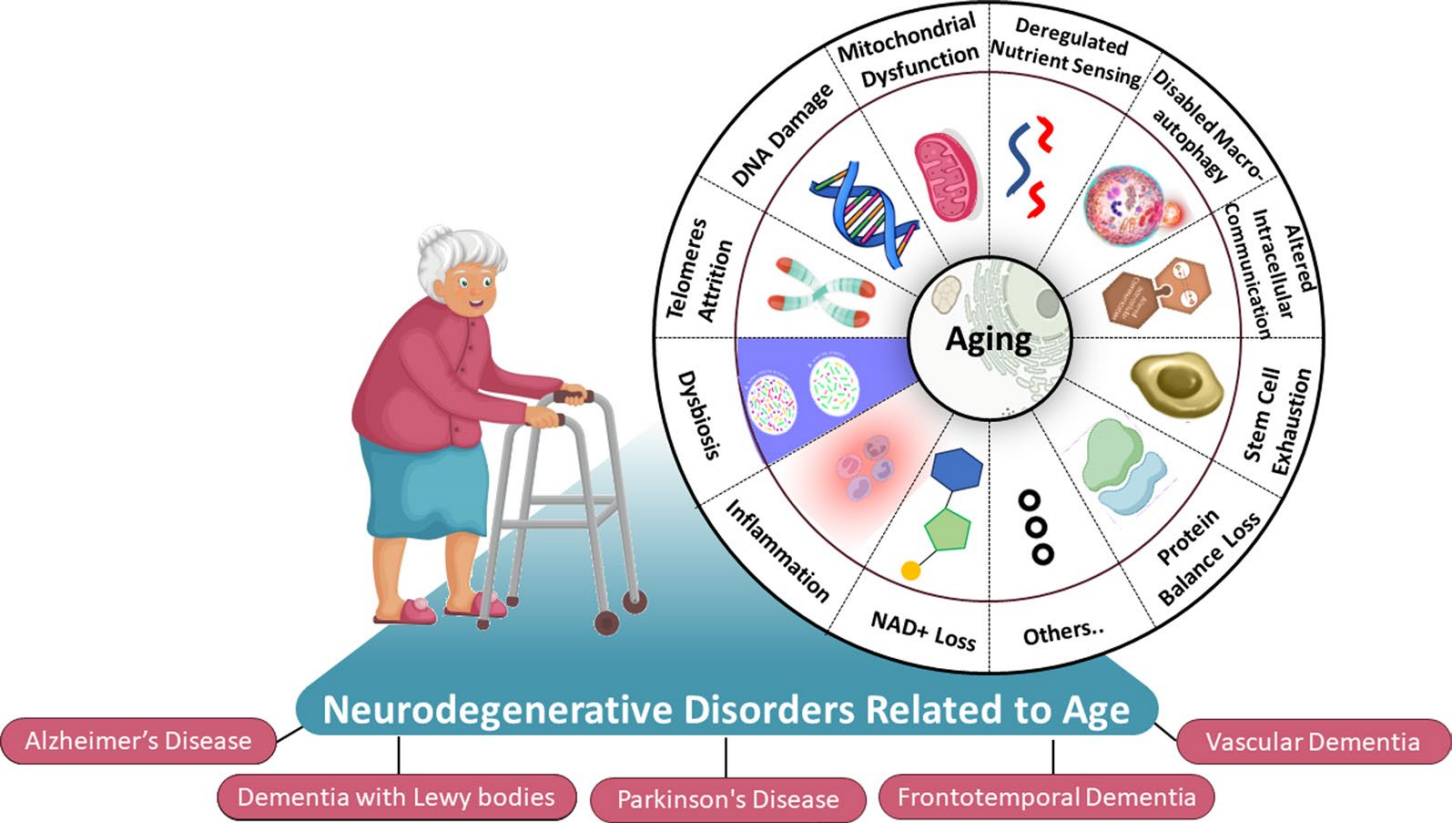
Drivers of aging and age-related diseases include several key physiological changes such as NAD + depletion, telomere shortening, mitochondrial dysfunction, stem cell exhaustion, impaired macro-autophagy, DNA damage, protein imbalance, inflammation, dysbiosis, deregulated nutrient sensing, and disrupted cellular communication. These aging-related features are fundamental, antagonistic, and interconnected, collectively promoting the aging process as aging progresses beyond a certain point, organ and tissue functions decline, leading to a higher incidence and mortality of neurodegenerative disorders related to age such as Alzheimer's disease (AD), Parkinson's disease (PD), frontotemporal dementia (FTD), dementia with Lewy bodies (DLB), and vascular dementia (VCID)

Aging is a progressive degenerative process that can be physiological and pathological [1–3] (Fig. 1). The physiological process of aging is triggered by a variety of biological and genetic processes, including telomere attrition [4, 5], DNA damage [6, 7], mitochondrial dysfunction [8, 9], reduced levels of nicotinamide adenine dinucleotide (NAD +) [10, 11], impaired macro-autophagy [12, 13], stem cell exhaustion [14, 15], inflammation [14, 15], protein imbalance [16], deregulated nutrient-sensing [17], altered intercellular communication [18–20] and dysbiosis [21, 22]. These changes collectively contribute to a decline in systemic function which are highly correlated with lifespan and incidence of all age-related diseases as shown (Fig. 1). In an aging world where people who are older than 65 are estimated to account for 20% of the population in 30 years. within that time range, it is predicted that the prevalence of neurodegenerative disorders related to age such as Alzheimer's disease (AD), Parkinson's disease (PD), frontotemporal dementia (FTD), dementia with Lewy bodies (DLB), and vascular dementia (VCID) will triple [23, 24]. There are currently few, if any, disease-modifying treatments (DMTs) available for these disorders, making dementia one of society's most burdensome conditions [25]. Abnormally folded protein aggregates gradually accumulate in neurodegenerative conditions of elderly population, such as AD, PD, and FTD, they interfere with network function leading to the eventual loss of certain neuronal networks [26, 27].

The literature search for this topic was performed using the PubMed, Embase, and Cochrane databases. The search followed a systematic approach and included articles from the inception of each database up to December 2023. Key search terms used were "aging", "neurodegenerative diseases", "Alzheimer's disease", "Parkinson's disease", "dementia", and "protein aggregates".

## Pathogenesis of Alzheimer’s disease

Alzheimer’s disease (AD), a serious age-related neurodegenerative condition, is the major reason for senile dementia [28]. Characterized by gradual cognitive decline, memory loss, language problems, and behavioral disturbances [29]. The etiology of Alzheimer’s disease has been the subject of numerous theories over the years for better understanding of the disease renowned hypotheses that explain the underlying pathogenic processes and may have an influence on therapeutic possibilities are the tau hypothesis and the amyloid deposition hypothesis [30]. The amyloid hypothesis depends on the aggregation of extracellular senile plaques containing misfolded amyloid-beta peptide (Aβ) [31, 32]. These plaques mediate inflammation and interfere with nerve signaling, which ultimately results in escalating the disease. In the brains of patients of AD and in animal models, local inflammatory reactions and uncontrolled astrocyte reactivity have been seen, and they are thought to be important contributors in the development and escalation of the condition. Nonetheless, they are not the main causes [33, 34]. On the other hand, the tau hypothesis indicates that tau protein is essential for preserving microtubules, which are critical for neuronal structure and function. Excessive phosphorylation of tau in AD causes it to separate from microtubules and form neurofibrillary tangles. This can affect axon transport and neuronal function, resulting in cognitive impairment in AD patients [30]. Numerous attempts to prevent the progression of the disease have been made throughout the years as a result of the excessive accumulation of misfolded proteins and neuroinflammation. Targeting misfolded proteins have been used in these attempts to decrease the plaque load [35, 36]. Or administrating systemic anti-inflammatory drugs to decrease brain inflammation. However, inconsistent and contradictory findings have been made, and no drug has yet to demonstrate effectiveness in restoring or limiting patients cognitive decline [37–43].

### Pathogenesis of Parkinson’s disease

PD is the second most prevalent neurodegenerative disease affecting old people, following Alzheimer’s [44, 45]. The incidence of Parkinsonism increases in the elderly, affecting populations over the age of 65 [46]. The motor symptoms of PD, such as tremors, bradykinesia, rigidity, as well as difficulties with walking and gait, are thought to be the outcome of pathological processes accumulation that exceed the ability of the brain to withstand or adapt to negative repercussions. It is becoming clear that the motor manifestations of PD could not appear for years have passed with continuing neurodegenerative cell death in the substantia nigra [47, 48].

The aggregation of alpha-synuclein protein (-syn) within neurons, which is coded by mutations in the -synuclein gene (SNCA), and the death of dopaminergic neurons in the substantia nigra are the hallmark features of PD [49, 50]. Later studies discovered that (α-syn) is apparent within Lewy bodies [51], which are intracellular protein aggregates primarily composed of (α-syn), neurofilament, and ubiquitin. The appearance of Lewy bodies in nerve cells is a hallmark of the pathology of PD [52] and is related to dopaminergic neuron death and activated microglia [53, 54]. In fact, (α-syn) can induce microglial activation and morphological changes [55].

### The role of microglia in the neurodegenerative disorder

There is evidence from pathology, genetics, and mouse studies that resident immune cells in the cerebrospinal nervous system play a significant function in disease advancement in both AD and PD. A variety of tissues have their own specialized, resident macrophages that perform both general innate immune performs and tissue-specific functions [56]. Microglia, the resident immune cells of the cerebrospinal nervous system, were previously a topic of much debate, but it is now accepted that they detect and respond to nearby pathogens. Microglia regularly survey their milieu, counter injury, and infectious agent, and contribute to tissue healing [57–59]. They also interact closely with nerve cells and can affect nerve cell function, because they are involved in formation of neurons de novo and synaptic pruning [57, 59, 60].

In a study, researchers noticed a subset of microglia with fragmented branches and beading in their process, which they termed dystrophic microglia [61, 62]. The number of dystrophic microglia was found to be higher in individuals with AD, DLB, or limbic predominant age-related TDP-43 encephalopathy compared to age-matched controls [63]. It is suggested that microglia with dystrophic morphology are senescent microglia [61]. When compared to activated microglia, senescent microglia are believed to have lower migration and phagocytic capabilities when compared to activated microglia [64].

## Current treatments for age-related neurodegenerative disorders

Numerous alternative therapies for age-related neurodegenerative disorders, including Alzheimer’s disease (AD) and Parkinson’s disease (PD), are presently under consideration and investigation. Stem cell therapy, gene therapy, and anti-aging drugs like metformin, rapamycin, resveratrol, and senolytics are some of those therapies.

### Stem cell therapy

The capacity to regenerate and differentiate into mature cells is what distinguishes stem cells from other types of cells. Exogenous stem cell implantation and stimulation/boost of endogenous brain progenitor cells are the two therapeutic approaches that use stem cells. Replacing damaged and lost cells directly, acceleration of tissue healing, growth molecules and trophic factors generation, immunomodulation and mobilization of endogenous stem cells, are a few potential methods of stem cell therapies. Engrafted stem cells have been shown to integrate into the local neuronal and synaptic networks to enable neurological regeneration in animal models of neurodegeneration [65–69].

### Stem cell therapies for Alzheimer’s disease

Mesenchymal stem cells (MSCs) start the remyelination, immunological balancing, and brain regeneration processes. Additionally, amyloidogenesis and/or microglial activation can be affected by MSCs by reducing the accumulation of amyloid-beta-peptide (Aβ) and promoting cognitive recovery. Adipose tissue-derived mesenchymal stem cells (AD-MSCs) can alleviate memory deficits by raising levels of IL-10 and vascular endothelial growth factor, regulating microglial activation in the brain, and lowering levels of amyloid plaques and Aβ in AD animal models [65, 70, 71].

### Stem cell therapy for Parkinson’s disease

Parkinson’s disease dementia (PDD) is characterized by cognitive impairment and the presence of α-synuclein. There is not much research assessing the effectiveness of transplantation of stem cells in treating PDD’s cognitive impairment, despite some reports suggesting that dopaminergic precursor transplantation reduced PD’s motor symptoms. Α-synuclein aggregates are regarded as harmful and cause neuronal deaths in various α-synucleinopathies. They are released by neurons through the process of exocytosis, which are subsequently absorbed by neuroglia and neuronal cells through the process of endocytosis. Additionally, it is hypothesized that Alpha-synuclein and *N*-methyl-d-aspartate (NMDA) receptor interaction may promote clathrin-dependent endocytosis of NMDA receptors [72–74].

### Gene therapy

Gene therapy consists of implanting a healthy gene into the genome to substitute for a damaged gene that causes a specific disorder. To implant the gene, a carrier which is known as a “vector” is used, the carrier must be very targeted and show efficiency in releasing one or more genes. That are the right sizes for clinical applications, not being acknowledged by the immunity, and are purified at high concentrations. Once the carrier has been inserted, it should not aggravate the immune system by causing any allergic or inflammatory reactions. It must improve regular functions, remedy deficiencies, and stop harmful activity. The inclusion of viral genetic material in the plasmid is a major aggravating factor, as it may trigger an immediate immune response in addition to a potential neoplastic transformation. There are currently two main methods for altering a cell’s genetic makeup: virus-mediated and by physical mechanisms using preparations made using advanced nanotechnology techniques [75–77].

### Gene therapy for Alzheimer’s disease

One strategy to lessen the pathogenesis of AD is to target Aβ. Zhang et al. produced a recombinant adeno-associated virus (AAV) that expressed the fusion protein CB-Abeta42 (cholera toxin B subunit and A42), which was one of the initial attempts. PDAPPV7171 transgenic mice developed large amounts of anti-A-42 antibodies after receiving a single intranasal, intramuscular, or oral dose of the AAV-CB-A-42 vaccine. High levels of anti-Aβ42 antibodies significantly decreased the amount of Aβ in the brain, reduced plaque-associated astrocytosis, and enhanced memory and cognitive performance [78, 79].

### Gene therapy for Parkinson’s disease

There have been found a number of potential genetic targets for the treatment of PD. Disease-modifying and non-disease-modifying targets fall under this category. In order to alleviate Parkinson’s manifestations, non-disease modifying therapies aspire to promote normalizing defective basal ganglia firing by expressing either dopaminergic or GABAgenic enzymes. These therapies have no effect on the fundamental pathophysiological process; they merely treat the symptoms. Treatment plans concentrate on avoiding PD-mediated cell death and/or rebuilding destroyed neurons to combat the disease. Nigral overexpression of growth factors has been proven to have neuroprotective qualities [80, 81].

### Anti-aging drugs

The physiological process of aging in the human body is irreversible, and the changes brought on by aging that accompany this process also have a role in several neurodegenerative diseases, such as AD and PD. Aging has become a major risk factor for most neurodegenerative diseases, which increases morbidity and death in elderly individuals. Anti-aging drugs including resveratrol, rapamycin, metformin, and senolytics (Table 1) are focused on the key ageing traits that affect autophagy and inflammation [82].

**Table 1:** Anti-aging drugs and their adverse effects

Drugs	Original use	Adverse effects
Metformin	Anti-diabetic	In people over 65, long-term metformin use raised the incidence of AD. It has been demonstrated that the reduction of mitochondrial respiration by metformin aids in the onset of PD. In a population-based study, it was discovered that it increased the rate of AD and decreased cognitive function in diabetic patients. In a cell culture model, it was discovered to increase the formation of Aβ, and the most common side effects in those who take it are diarrhea, nausea, flatulence, indigestion, vomiting, and abdominal discomfort. Metformin use for a long time caused a vitamin B12 deficit [83]
Rapamycin	Immunosuppressant, anticancer	Hyperlipidemia, hypercholesterolemia, hypertriglyceridemia, glucose intolerance, insulin resistance, new-onset diabetes, anemia, thrombocytopenia, dermatological events, gastrointestinal disorders, sinusitis, respiratory, urinary infections, and testicular dysfunction [84]
Resveratrol (RE)	Anti-inflammatory, antioxidant, anti-platelet, anti-hyperlipidaemic, immune-modulator, anti-carcinogenic, cardioprotective, vasorelaxant, and neuroprotective effects [85]	White blood count (WBC), IL-6, tumor necrosis factor (TNF) levels, and alanine aminotransferase (ALT) levels, can all be severely impacted. High doses of RE might cause nausea, mild to moderate diarrhea, anal pruritis, and allergic reactions [85]
Senolytics	Anti-cancer drugs	Aging pancreatic beta cells have demonstrated enhanced release of insulin, and elimination of these cells may as a result induce an imbalance in the homeostasis of glucose. Additionally, animals treated with navitoclax showed cytotoxicity targeting bone marrow precursor cells and osteoblasts [86]

## Novel approaches to the treatment of age-related neurodegenerative disorders

In order to regulate the immune response and promote self-tolerance, immunological checkpoints such as the regulatory molecules programmed cell death protein-1 (PD-1) and its ligands are fundamental. There are two identified ligands for PD-1: PD-L1 (additionally known as B7 homolog 1, B7-H1) and PD-L2 (or B7-DC) [87]. When triggered by PD-L1 and PD-L2, PD-1 inhibits T cell activity. Adaptive and innate immune cells, mesenchymal cells, and cancer cells are just a few of the cell types for which PD-L1 expression can be induced. The PD-L2 protein, on the other hand, is only produced by a very restricted subset of tumor cell types and antigen-presenting cells (APCs) [88]. T cell exhaustion, a potentially persistent state of malfunction, is caused by PD-1 and PD-L1 interaction. Tumor cells take advantage of this interaction to escape immunosurveillance. Immune checkpoint inhibitors (ICIs) have become a crucial component of cancer treatment [89], including melanoma, non-small-cell lung cancer (NSCLC), small cell lung cancer (SCLC), primary mediastinal large B-cell lymphoma (PMLBCL), classical Hodgkin lymphoma (cHL), head and neck squamous cell carcinoma (HNSCC), colorectal cancer (CRC), renal cell carcinoma (RCC), hepatocellular carcinoma (HCC), bladder cancer, Merkel cell carcinoma (MCC), and microsatellite instability high (MSI-H) or DNA mismatch repair deficient (dMMR) adult and pediatric solid tumors [90]. The host immune system is induced by immune checkpoint inhibitors to fight cancer cells [91, 92]. By boosting chemokine-dependent immune cell infiltration into the malignant disease zone and stimulating IFN-γ at the tumor tissue, blocking the PD-1 pathway could enhance fighting chances against cancer [93, 94]. By inhibiting the production of glycolysis-related enzymes, stifling glucose absorption, and lowering AKT phosphorylation, anti-PD-L1 antibodies can have a detrimental effect on cancer cell metabolism [95, 96]. Therefore, new and innovative approaches to treating neuroinflammation are therefore made possible by immunotherapy and immune checkpoint blockade.

## The general role of PD-1 and PD-L1 in immune system

**Fig. 2:**
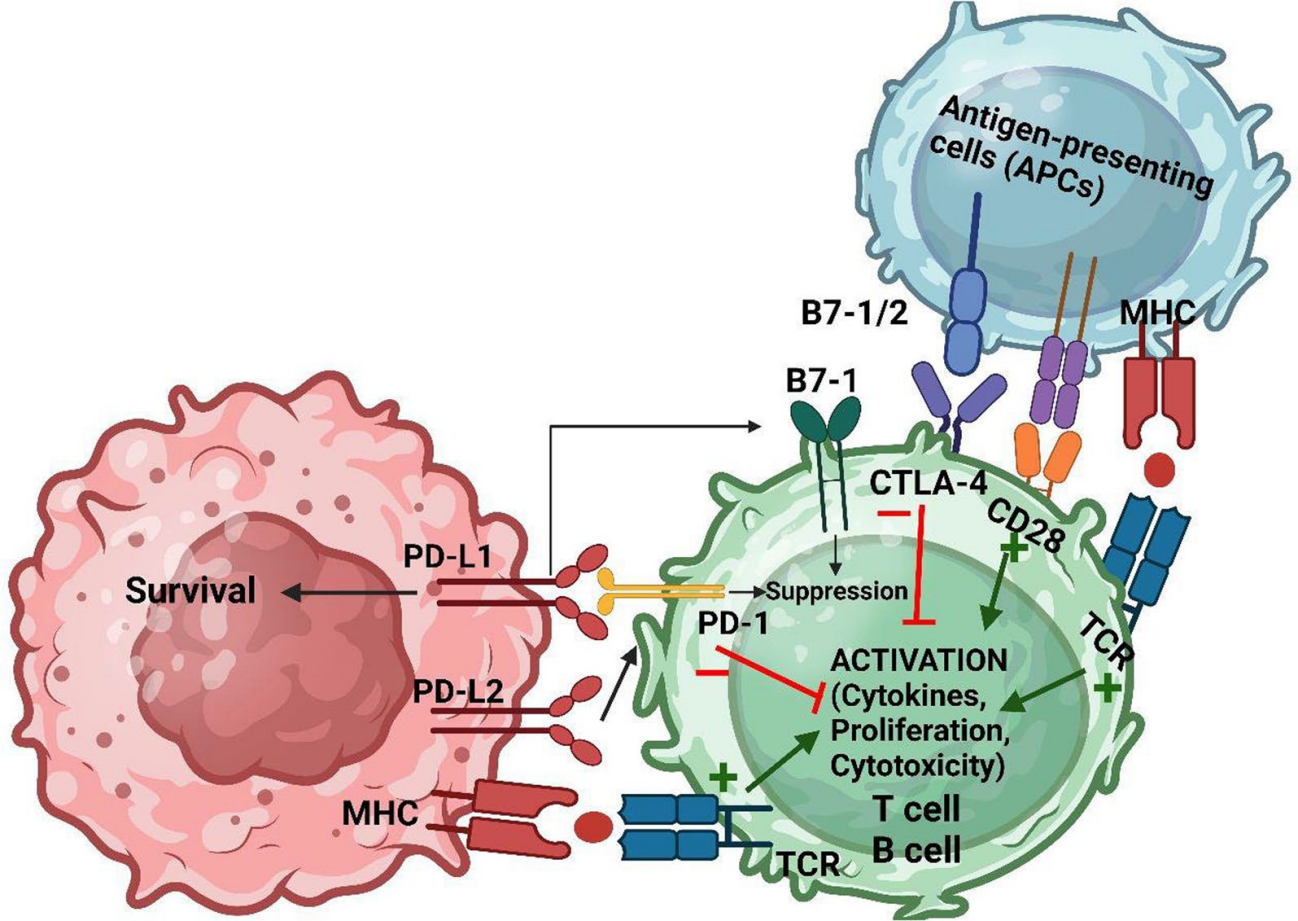
Understanding T lymphocyte stimulation and the PD-1/PD-L1 pathway: the pathway of programmed cell death (PD) and cytotoxic T lymphocyte antigen-4 (CTLA-4) plays a crucial role in the suppression of T-cell activation. T-cell activation is stimulated by the interaction between the major histocompatibility complex (MHC) and the T-cell receptor (TCR), along with the co-stimulatory interaction between B7-1/2 and CD28. In contrast, CTLA-4 binds to B7-1/2 and delivers an inhibitory signal to prevent T-cell activation. Within the PD pathway, there are interacting molecules: PD-1, PD-L1, PD-L2, and B7-1 (CD80). PD-L1 and PD-L2 are ligands for PD-1, and their binding to PD-1 results in the suppression of T-cell activation. Additionally, PD-L1 interacts with B7-1 (CD80) on activated T cells to inhibit their activity

A healthy immune response is essential for bodily protection while avoiding host injury. T lymphocytes stimulation needs two indications, as is widely known. The initial signal is produced as a result of the interplay of the T lymphocyte receptor (TCR) in hand with its corresponding antigen, which is introduced by molecules of MHC generated by antigen-presenting cells (APCs). The engagement of receptors that are responsible for co-signaling on T lymphocytes with declared ligands on APCs provides the second signal. Those molecules of co-signaling may induce both suppressing and stimulating stimuli, therefore the balance of these signals determines the fate of T cell activation. The system of pathways that include co-signaling permits the body’s immune system to fine-tune immunological response control. PD-1 (sometimes referred to as CD279) is a receptor that causes cell death. major receptor of coinhabiting on surface of the CD28 family that inhibits T and B lymphocytes induction via interactions with its PD-L1 (which is additionally recognized as CD274 or B7-H1) and PD-L2 (also known as CD273 or B7-DC) ligands [97–101]. PD-1 is critical in the regulation of T lymphocytes exhaustion and induction. Extreme activation of antigen of T lymphocytes, as in infection chronically or cancers, promotes CD8 and CD4 T lymphocyte fatigue, which is featured by losing T lymphocyte effective abilities such as influenced growth, suppressed cytotoxicity and expression of transcription factor as shown (Fig. 2) [102, 103]. PD-1 ligation shall also limit the formation of effector T cells by switching through glycolysis regarding ß-oxidation of fatty acid, the me

tabolic makeup of excited T lymphocytes [104]. Few studies found the fact that the interplay of PD-1, PDL-1 and PDL-2 modulates peripheral CD8 and CD4 T lymphocyte resistance immunologically by attenuating self-reactive T lymphocyte reaction during self-antigen presentation by dendritic cell (DC) [105, 106]. Also, some studies have found interesting that the two PD-L1 along with PD-1 are strongly displayed on regulatory T lymphocytes [107, 108]. On the other hand, effective T lymphocytes, where the signaling of PD-1 limits growth, PD-1 stimulation manages Treg lymphocyte growth, maintenance, and function [108]. PD-1 as well as PD-L1 are both substantially expressed on Treg cells, according to other studies. Unlike effective T lymphocytes, where PD-1 alerting reduces multiplication, PD-1 stimulation regulates a developing effect. Treg lymphocyte regulates a sustaining effect and abilities, for instance, PD-L1 transformed mice naive T lymphocytes carrying CD4 to regulatory T lymphocytes by inhibiting the Akt/mTOR and ERK2 cascades while simultaneously increasing the phosphatase and tensin homolog (PTEN) [109–113]. According to two studies, the interplay between PD-1 and its ligands modulates T lymphocyte tolerance with the peripheral CD4 and CD8. Self-reactive T cell response is reduced when DC presents self-antigen. Surprisingly, PD-1and PD-L1 axis are important in modifying regulatory T (Treg) cell development and function. FoxP3 expression in inducible Treg cells requires signaling by PD-L1 shown on DCs in spleen. DCs lacking PD-L1 had a significantly reduced ability to convert naive antigen-specific T lymphocytes having CD4 receptors into regulatory T lymphocytes in vitro [114]. In addition, traditional polarized Th1 human T lymphocytes displaying PD-L1 that were turned into regulatory T lymphocytes with tolerance, showing a decrease in Tbet, an increase in the production of IFNs, and a rise in the conveying of FoxP3. By this scenario, PD-L1-dependent Th1-to-regolatory T lymphocytes division was caused by decreased Th1 lymphocytes STAT stimulation via the route of SHP1 and 2 communications, which is downregulation of the receptor of PD-1 [115]. It has lately been demonstrated that the signals to PD-1 preserve equilibrium of Foxp3 in regulatory inducible T lymphocytes by blocking asparaginyl endopeptidase (AEP), an endolysosomal protease that immediately breaks Foxp3 in those lymphocytes [116].

## The potential role of PD1/ PDL1 as “immune regulators”

PD1/PDL1’s proposed function as “immune regulators”. PD-1 is an essential immunosuppressive molecule that may regulate T-cell effector activities in a variety of ways. It has been extensively demonstrated that PD-1 receptor signaling suppresses TCR-mediated cell activation (for instance, division and cytokine synthesis) [117–119]. Nevertheless, recent research indicates that the PD-1 axis is also essential for managing T-cell activation throughout various neuroinflammatory disease models. Mair and al. examined a role of PD-1 within both the growth and preservation of T-cell flexible tolerance [for example, once antigen activation proceeds in vivo, T cells enhance a hyporesponsive state [120]. After being in an EAE approach, participants were exposed to substantial quantities of autoantigens. That study discovered that the presence of PD-1 is higher on adaptive T cells with their CD4 receptors in the CNS. However, PD1 removal did not hinder T cells are prevented from forming an adjusted phenotype in living cells as well as in laboratory, thus PD-1 is not essential for T cells to keep being nonresponsive in EAE [120]. This data indicates that PD-l is not required for that process, the contradiction of most earlier data on PD-1 participation in T-cell adjustment [121]. It was revealed utilizing an identical disease model for animals (EAE) that T lymphocytes with their CD4 accessing the CNS throughout the pinnacle of sickness stimulate PD-1, and that increase is linked to a decrease in Th1 cells as a result of PD L1 + microglia producing nitric oxide. These findings support PD-1’s function in EAE control by suppressing transformation of Th1 cells [122]. The reduction of T-cell with CD8 receptors activity observed in rats with SCI was linked to the activation regulating processes, consisting of overexpression of PD-1 along with PD-L1. Wounded rats showed a greater quantity of PD-1 and T-cell with CD8 receptors, in addition to a greater surface transcription of this receptor. The overproduction of PD-1 ceases the immune-inflammatory process and prevents inflammation from spreading at the site of harm [123]. Myeloid-like cells are called microglial cells. Live in the CNS and are highly reactive to variations in brain homeostasis. During autoimmune inflammation, microglial cells act as the point of contact involving the body’s immune system and the CNS [62]. In healthy mice, microglial cells produce PD-L1 at low levels (about 20%), yet after 1 week of viral brain infection, the expression level could rise to more than 90% [124]. In the course of neuroinflammation, both microglia and astrocytes boost PD-L1 in combination with MHC I and MHC II, indicating that indigenous glial cells have a role in reducing CNS illness [125]. Furthermore, adoptive transfer of PD-L1-expressing M2-polarized microglia decreased. The seriousness of an existing EAE, underlining the significance of PD-L1’s regulation in neuroinflammation [126]. However, using the PD-L1 recombinant protein construct to activate PD-1/PD-L1 signaling significantly decreased inflammatory responses and cerebral edema following SBI. Therefore, in SBI, the PD-1 as well as PD-L1 signaling mechanism shall be implicated as part of a “self-protection function” [127]. The most prevalent is Alzheimer’s disease, a kind of dementia in humans distinguished by symptoms such as mental impairment as well as neuronal degeneration. AD diseases are distinguished because of two circumstances: extracellular amyloid plaques (A) in addition to neurofibrillary tangles that contain (Tau) intracellularly [128]. The biological process behind TAU protein and amyloid buildup is unclear, as well as the system’s defenses might be involved in the development of pathogens [129]. According to recent studies, systemic immunity ought to be enhanced instead of being stifled in order to aid brain recovery via an immune-dependent reaction. The PD-1 route has been hypothesized in this regard. A viable Alzheimer’s disease targeted treatment [130]. The transfer of blood-borne myeloid-like cells (monocyte that are derived from macrophages) to the CNS is currently proven in Alzheimer’s disease rat models to be beneficial neurologically. PD-1 on T lymphocytes, as well as PD-L1 on monocytes and macrophages, are considerably lower in AD patients and persons with moderate memory impairment when compared with time and gender-matched healthy controls [131]. The PD-1/PD-L1 axis represents a prospective target for AD therapy using antibodies.

### The potential of PD-1/PD-L1 ICIs combination therapy in treating Alzheimer’s disease with muramyl dipeptide

**Fig. 3:**
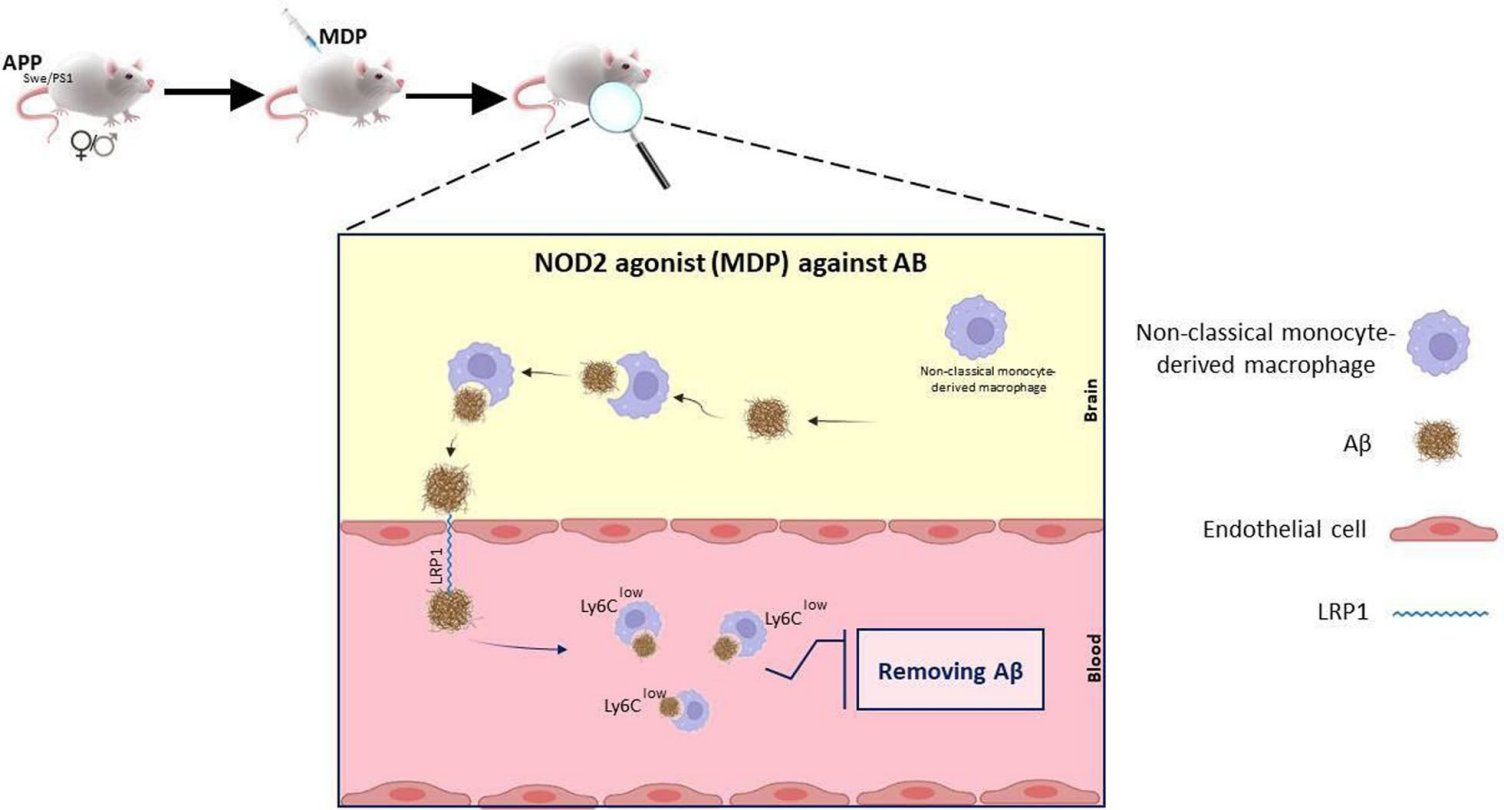
The well-known strain APPswe/PS1 was administered chronically with MDP once a week for three months. The findings show how a NOD2 agonist triggers the transformation of inflammatory monocytes into patrolling monocytes. The toxic vascular Aβ is then enveloped as these cells patrol the arterial network to remove it from the brain. MDP aids in the removal of Aβ from the brain and into the blood, where it might be collected by blood monocytes that are on the lookout, by activating LRP-1 transporters [91, 94]

A synthetic immunoreactive peptide called muramyl dipeptide (MDP) is composed of *N*-acetyl muramic acid and a short amino acid chain of l-Ala-d-isoGln that was detected by the human innate immune system, which is a cytoplasmic receptor called nucleotide-binding oligomerization domain 2 (NOD2). It can activate the immune system by increasing the production of IFN-γ and other cytokines, promoting the differentiation and proliferation of a specialized group of the WBCs called lymphocytes which are crucial for the immune system’s defense against foreign bodies. Additionally, it may be used for increasing the potency of vaccines and drugs as an effective adjuvant [132, 133]. MDP can produced synthetically despite it being naturally derived from Gram-negative and Gram-positive bacteria cell walls [134]. The modulation of the innate cells of the immune system, such as the local patrolling monocytes and microglia, has been studied recently in relation to the NOD2 receptor ligand MDP. Preclinical results show that MDP injections can delay cognitive decline and protect female and male mice’s blood–brain barrier (BBB) by different mechanisms as presented in Fig. 3. For example, the findings in male mice support the idea that the MDP activates the BBB’s sink effect, reducing the brain’s accumulation of amyloid. Based on osmotic principles, this hypothesis states that amyloid is in balance with blood vessels and the brain, and the objective is to eliminate amyloid from the periphery and expel it from the brain. MDP appears to have a more limited beneficial effect on female brains because microglia encircle amyloid plaques and prevent amyloid peptides from spreading [135]. Accordingly, investigations have shown that monocyte populations can do this by removing amyloid plaques and producing growth factors and anti-inflammatory compounds, which causes the sink effect [136]. Simultaneously, an integrated approach that combines modulation of NOD2 and PD-1 could yield superior outcomes, according to recent evidence. This approach provides the transformation of inflammatory monocytes into patrolling monocytes. The promise of these therapeutic interventions is being understood by pharmaceutical entities who are currently conducting preclinical research on them. In summary, there is strong evidence suggesting that MDP has the potential to be a catalyst for this monocyte discussion, and the PD-1 inhibitor could trigger the patrolling monocytes in the brain [137].

### PD1/PDl-1 from experimental to clinical investigation

Immunotherapy focuses on reducing the damaging impact of protein accumulation such as Aβ and αSyn accumulation by removing and neutralizing toxic species. Results from the first immunization experiment against Aβ demonstrate that a small number of meningoencephalitis patients that changed the vaccine’s formulation led to a change in the field of immunotherapy from active to passive immunization. The field has recently advanced to target other proteins in AD, specifically “Syn than Aβ” [138].

## MAbs in neurodegenerative disorder

Monoclonal antibodies (mAbs) are synthetic molecules designed to act as mimic antibodies, capable of either replenishing, improving, or replicating the immune system's assault on cells. These antibodies are monospecific, showing that they are identical and originate from a single type of immune cell, all of which are clones of a different parent cell [139]. There are multiple categories of mAbs. Firstly, there are naked mAbs, which operate independently without any attached drugs or radioactive substances. Secondly, there are conjugated mAbs, which are linked to chemotherapy drugs or radioactive particles to directly administer these treatments to specific cells. Lastly, there are bispecific mAbs, which are designed to bind to two different antigens simultaneously, thereby enhancing their capacity to bring together target cells and immune cells [140, 141]. Monoclonal antibodies (mAbs) are under investigation in neurodegenerative disorders like Alzheimer's and Parkinson's disease for their potential to target and neutralize pathogenic proteins that contribute to disease progression. In the case of AD, mAbs such as aducanumab are used to specifically target Aβ plaques. The goal is to decrease the accumulation of these plaques and maybe slow down the decline in cognitive function [142]. In the case of Parkinson's disease, researchers are studying the use of monoclonal antibodies to specifically target alpha-synuclein, a protein that forms damaging aggregates in the brains of people with the condition [143]. Multiple clinical trials have been carried out to assess the effectiveness and safety of these drugs. The FDA controversially approved aducanumab based on the evaluation of its effectiveness in Alzheimer's patients during the EMERGE and ENGAGE trials [144]. Ongoing trials in Parkinson's disease are investigating the use of antibodies, including prasinezumab. Early results indicate that these antibodies have the potential to slow down the progression of the disease [143].

### Aducanumab in Alzheimer’s disease

Aducanumab, also known as Aduhelm®, is a monoclonal antibody specifically developed for the treatment of AD. The mechanism of action involves specifically targeting amyloid-beta plaques, which are a characteristic feature of this disease. Aducanumab has the ability to bind to clustered amyloid-beta and facilitate its elimination from the brain, which could delay the advancement of the disease. Clinical studies had various results, but the FDA's approval in 2021 via fast track was significant. Discussion developed due to concerns regarding the decision's efficacy and the need for further validation of clinical benefits. Aducanumab represents a significant advancement in the treatment of AD, effectively targeting a crucial pathology associated with this fatal neurodegenerative condition. The safety and effectiveness of aducanumab in AD patients were evaluated in two significant Phase 3 clinical trials called ENGAGE and EMERGE. To assess the efficacy of aducanumab in reducing amyloid-beta plaques and potentially slowing down cognitive decline, these trials enrolled individuals diagnosed with early-stage AD. The EMERGE trial yielded encouraging results, demonstrating a significant reduction in amyloid-beta plaques and interesting implications for cognitive and functional outcomes in clinical settings. The ENGAGE experiment has been discontinued due to a lack of comparable favorable outcomes. Despite the inconsistent results from the two trials, the evidence provided by EMERGE was sufficient to justify aducanumab's application for approval by the FDA based on its effectiveness. The trials underscored the challenges in developing efficacious therapies for AD and the intricate and diverse nature of treatment results [144, 145] (Table 2).

**Table 2:** Some clinical trials of aducanumab in Alzheimer’s disease

Drug	Target	Mechanism of action	Clinical Trial.gov	Phase	Intervention	Status	Refs.
Aducanumab	Aβ	Target and bind to Aβ plaques in the brain. Then facilitates clearance of Aβ through activation of the immune system, specifically microglia, which phagocytose the antibody-bound Aβ aggregates	NCT05216887	Phase 1	Aducanumab single IV weight-based dose on day 1 or two fixed SC injections on day 1 and 15	Active, not recruiting	[146]
			ENGAGE, NCT02477800a, 221AD301, 2015–000966-72	Phase 3	Monthly IV infusions of aducanumab (low or high dose) or placebo	Terminated (study was discontinued based on futility analysis done and not based on safety concerns. Follow-up visits and closing out study activities are completed)	[144, 147]
			EMERGE, NCT02484547a, 221AD302, 2015–000967-15				[144, 147]
			ENVISION phase IV confirmatory trial, NCT05310071a	Phase 4	Monthly IV infusion of aducanumab up to 10 mg/ kg or placebo	Recruiting	[148, 149]
			EMBARK, NCT04241068a, 221AD304, 2019–004368-22	Phase 3	10 mg/kg of aducanumab by IV infusion every 4 weeks for a total duration of 100 weeks	Active, not recruiting	[150]

### Lecanemab in Alzheimer’s disease

In january 2023, lecanemab (Leqembi®) (also known as BAN2401) is approved by the FDA for the treatment of AD to be initiated in early AD (mild cognitive impairment [MCI] due to AD or mild AD dementia) with confirmed brain amyloid pathology. It is a monoclonal antibody that targets Aβ aggregates implicated in AD pathology. Derived from a blood lymphocyte library of elderly individuals without cognitive impairment, Lecanemab specifically binds to protofibrils and oligomers of Aβ, potentially reducing their toxic effects on neurons and slowing disease progression. The clinical development of Lecanemab has shown promise, with early trials suggesting it may help reduce Aβ plaques and possibly alleviate cognitive decline in Alzheimer's patients [151]. In a double-blind, phase 3 trial, compared to placebo, lecanemab significantly reduced brain amyloid levels and moderately slowed cognitive and functional decline in early AD over 18 months (Clarity AD ClinicalTrials.gov number, NCT03887455; funded by Eisai and Biogen). The work brought attention to the fact that Lecanemab is very selective for harmful amyloid species. The lecanemab group did have a higher incidence of adverse events, but these were often moderate and asymptomatic, especially ARIAs (amyloid-related imaging abnormalities) [151, 152].

### Other MAbs used in Alzheimer’s disease

Additional monoclonal antibodies used in the treatment of AD include gantenerumab, donanemab, solanezumab, crenezumab, and tilavonemab. Each of these antibodies serves specific functions in addressing the pathophysiology of the illness, accompanied by certain adverse effects and distinctive benefits.

*Gantenerumab* specifically attaches to aggregated Aβ plaques, aiding in their removal of Aβ from the brain. This action has the potential to decrease the amount of plaque and enhance cognitive function. One of its benefits is its high effectiveness in lowering amyloid plaques, which are a significant characteristic of Alzheimer's disease. Nevertheless, the potential adverse consequences of this treatment could include ARIA, characterized by cerebral edema and microhemorrhages in the brain [153].

*Donanemab* targets the pyroglutamate-modified type of Aβ, which is known for being very toxic, to make it easier for the body to get rid of it and maybe slow the disease's progression. The main benefit of this approach is its ability to specifically target a highly toxic type of Aβ, perhaps leading to more efficient elimination of plaques and protection of neurons. Like other antibodies that target Aβ, it can also induce ARIA, resulting in adverse effects such as cerebral edema and microhemorrhages [154].

*Solanezumab* is a humanized monoclonal antibody that targets the mid-domain of the Aβ peptide. It makes it easier for the brain to get rid of soluble Aβ and slows down neurodegeneration. Preclinical studies have shown that it creates strong connections with Aβ, facilitating the transfer of Aβ from the brain to the plasma. This has resulted in enhanced cognitive performance in transgenic mouse models of Alzheimer's disease [155]. It attempts to inhibit the aggregation of soluble Aβ monomers into plaques. Also, it offers the benefit of a more advantageous safety profile, which typically leads to a lower incidence of serious adverse effects in comparison to other antibodies. Common side effects of this treatment may include moderate infusion-related symptoms such as chills, fever, and rash. However, the effectiveness of this treatment has shown inconsistent outcomes in clinical studies [156].

*Crenezumab* targets different forms of Aβ, including monomers, oligomers, and fibrils, to get rid of amyloid aggregates and lower neurotoxicity in a lot of different brain diseases. Crenezumab has a benefit due to its comprehensive targeting approach, which can address all phases of amyloid disease. The adverse effects of this medication are generally moderate; however, they may include ARIA, headaches, and infusion-related symptoms such as nausea and exhaustion [157].

In Alzheimer's disease, *tilavonemab* specifically targets the tau protein, which is responsible for the formation of neurofibrillary tangles. It aims to decrease tau pathology and the resulting neurodegeneration. Tilavonemab has a distinct advantage in its specific targeting of tau protein, which offers a complementary strategy to medicines that target Aβ. The typical adverse effects of this medication may include responses connected to the infusion process and headaches. However, since it is still being studied, its overall safety profile has not been fully determined [158]. Tilavonemab has shown satisfactory tolerability but did not exhibit efficacy in the treatment of early-stage Alzheimer's disease. Consequently, it is not advisable to conduct additional studies on tilavonemab for this particular disease [159, 160].

These monoclonal antibodies exemplify the diverse strategies being explored to tackle the multifaceted nature of Alzheimer's disease, each with its own mechanism of action, associated side effects, and specific advantages.

### Monoclonal antibodies targeting α-syn in Parkinson’s disease

PD, DLP, MSA, and a percentage of AD are neurological diseases characterized by synucleinopathies which is an accumulation of α-syn in cortical and subcortical areas of neuronal and non-neural cells [51, 161–163]. The unique strategy for getting rid of the buildup senile particles from the brain using antibodies was logical, given that Aβ aggregates are located in the extracellular matrix (ECM) [35]. How this strategy could be applied to DLB, PD, and other synucleinopathies was unclear, as a substantial amount of Since a significant amount of proteinaceous aggregates were believed to deposit intracellularly within the neural cells of the striatonigral system, limbic areas, and deep layers of the neocortex which reflects an imbalanced proteostasis network (PN) causing neurological inflammation and failure of the synapses caused by neurodegenerative oligomer production and cell-to-cell transmission of oligomers, protofibrils, and fibrils [161, 164–166]. Nonetheless, immunizing that recently developed α-syn transgenic mice conjugated with human alpha-synuclein and Freund adjuvant resulted in the formation of elevated titer antibodies against C-terminal α-syn which were able to eliminate αlpha-syn accumulation in neural cells and alleviating functional deficits and neurodegeneration [167] via additional immunization experiments both active and passive in transgenic, viral, and injection of preformed fibrils (PFFs) [168, 169]. These antibodies act in a variety of ways, including detecting α-syn clumps in the membrane and inducing endocytosis and clearance through autophagy, lysosomal activity, or proteasomal destruction [170, 171]. Additionally, it is possible for antibodies to move within cells. With single-chain antibody fragments (scFvs) or intrabodies particularly designed to pass through the plasma membrane via apolipoprotein B (ApoB), trans-acting activator of transcription (TAT) fusion proteins, or by utilization of endogenous agonist [172, 173]. A substantial body of evidence indicates that α-Syn is not just a marker for Parkinson's disease (PD) but actively contributes to its progression [161]. Potential treatments for synucleinopathies involve reducing the accumulation of α-Syn both intracellularly and extracellularly or enhancing its clearance, which may serve as effective therapeutic approaches for Parkinson's disease (PD) and related disorders [174, 175]. The discovery of disease-causing α-Syn species and their ability to spread between cells has created new avenues for developing therapies intended to delay or stop disease progression [174]. These strategies typically involve: (1) immunization; (2) reducing the production of α-Syn protein; (3) inhibiting α-Syn oligomerization; (4) decreasing intracellular or extracellular α-Syn aggregation; (5) enhancing α-Syn clearance; (6) targeting genes/proteins that influence α-Syn aggregation or processing (e.g., silencing SNCA); (7) increasing the breakdown of α-Syn by proteolytic enzymes; (8) minimizing posttranslational changes as oxidation, nitration, phosphorylation, and C-terminal cleavage [73, 174, 176]. While these treatments show promise, many of these therapies are still in early research phases and need more clinical studies to confirm their safety and effectiveness in treating α-Syn-related disorders. Immunotherapy that targets extracellular α-Syn transmission remains crucial from a clinical standpoint [177]. In theory, antibody-based therapies are the most effective treatments for targeting pathogenic proteins, especially those located in the extracellular environment [178, 179]. On the other hand, various preclinical and clinical studies have developed alternative targeting methods to indirectly slow the spread of α-Syn (Tables 3 and 4) [138]. These approaches have long-term therapeutic promise since they restrict the extracellular release of aggregated α-Syn and prevent the propagation of pathogenic proteins, resulting in neuroprotection in PD patients.

**Table 3:** Active and passive immunization in Parkinson's disease (PD) animal models

Name	Antibody	Model	Route of administration	Target site	Time and dose	Effect of α-Syn	Effect on behavior	References
PDO1A AFFiRiS	AFF	PDGF, mThy1, MBP α-Syn mice	s.c	C-terminus	8 months; 80 μg/mL, 100 μL	Decreased α-Syn accumulation prevented α-Syn propagation	Motor improvements: decreased errors on round beam test	[180, 181]
UB-312 Vaxxinity	UB-312	Thy1SNCA/15 mice (line 61)	i.m	C-terminus	15 months; 40 µg per injection	Decreased levels of α-Syn oligomer	Enhancements in motor skills, learning, and memory: decreased errors on the round beam test	[170, 182–185]
Prasinezumab (PRX002)	9E4	PDGF, Thy1 (line D, 61)-α-Syn mice	i.p./i.v	C-terminus	6 months; 10 mg/kg	Decreased DA cell loss Decreased Calpain-cleaved α-Syn oligomer Decreased α-Syn accumulation	Motor performance: increased Cylinder, Pole, and Rotarod test	[170, 171, 181]
TAK-341	MEDI1341	Rat and cynomolgus monkey	i.v	C-terminus	13 weeks; from 3 mg/kg to 100 mg/kg; 25, 75, and 150 mg/kg	Decreased contralateral and ipsilateral α-Syn Decreased Interstitial and CSF fluid α-Syn levels Decreased α-Syn spread along axons	–	[186]
Cinpanemab	BIIB054 (NI-202)	Rat and cynomolgus monkey. A53T (M83), BAC A53T α-Syn PFF	i.p./i.v	N-terminus	2–4 months; 10 mg/kg and 30 mg/kg	Decreased α-Syn pathology maintained and improved DAT levels	Motor improvements: increased latency in time to fall in wire hang test	[187, 188]
Syn303	–	wt mice PFF	i.p	N-terminus	180 days; 30 mg/kg	Blocked α-Syn spreading Decreased DA cells loss Decreased insoluble α-Syn aggregation and pS129-α-Syn	Increased latency to hang (wire-hang time)	[169]
Lu AF82422	–	Rat and cynomolgus monkey	i.v	C-terminus	4 weeks, 1 dose; 0–600, 0–300, and 1–30 mg/kg	Decreased α-Syn aggregation	–	[189]
1H7, 5C1	α-Syn antibodies 1H7 5C1 5D12	Thy1 α-Syn (line 61) mice	i.p	C-terminus	6 months; 10 mg/kg	Decreased TH loss in striatum (35%) Decreased α-Syn accumulation Decreased synaptophysin + MAP2 decreased astro and microgliosis	Decreased memory and learning deficits Decreased Error on transversal beam Decreased Memory and learning deficits	[181]
AB1	Syn303	Nigral AAV-CBA α-Syn in wt rats	i.p	N-terminus	3 months; 1 mg/rat (2 × first), 0.5 mg/mL	Decreased α-Syn in SN Decreased α-Syn brain homogenate Decreased DA and NeuN cells loss	Motor improvement: increased on cylinder test	[190]

**Table 4:** Immunotherapeutic agents undergoing clinical trials for Parkinson's disease

Drug	Target	Mechanism of action	Phase	Clinical Trials.gov	Status	Results	References
Prasinezumab (PRX002)	C-terminus	Aggregation α-Syn at AA 118–126	Phase II	NCT03100149	In progress, not recruiting	Safe and easily tolerated Decreased free serum α-Syn	[191, 192]
Cinpanemab (BIIB054)	N-terminus	Aggregation, fibrillar α-Syn	Phase I	NCT02459886	Completed	Safe and tolerable with no TEAEs	[193]
Cinpanemab (BIIB054)	N-terminus	Aggregation, fibrillar α-Syn	Phase II	NCT03318523	Terminated	The study failed to fulfill its intended outcome	[194]
MEDI1341/TAK-341	C-terminus	Monomeric and Aggregation α-Syn at the AA 103–129 region	Phase I Phase III	NCT04449484 NCT05526391	Completed Recruiting	Result pending	[186]
UCB7853	C-terminus	Aggregation α-Syn	Phase I	NCT04651153	In progress, not recruiting	In progress	–
PD01A AFF	C-terminus	Peptide-based vaccine	Phase I	NCT01568099	Completed	Good tolerability and safety of s.c. administration	[195]
PD03A AFF	C-terminus	Mimics α-Syn peptide vaccination acts as B cell epitope	Phase IB Phase I	NCT02618941 NCT02267434	Completed, result pending Completed	Safe and well tolerated	[196]
UB-312	C-terminus	Targets oligomeric and fibrillary α-Syn	Phase IA and B	NCT04075318	Completed	Safe, easily tolerated, and induced anti-α-Syn antibodies in serum and CSF	[197]

## Limitations of immune checkpoint blockade in age-related neurodegenerative disorders

Because the brain is an immune-privileged organ, it contains defenses against the immune system’s attacks on healthy cells, immune checkpoint inhibitors find it challenging to enter the brain and find the appropriate cells [198]. Immune checkpoint inhibitors might not be able to identify and target the correct cells, even if they do enter the brain [199, 200]. This is due to the possibility that the immunological checkpoint molecules expressed by cells engaged in neurodegenerative diseases differ from those expressed by cancer cells [201, 202]. The goal of targeting immune checkpoints in cancer is to inhibit checkpoints that avoid the immune system from attacking tumor cells, whereas the goal of targeting immune checkpoints in neurodegenerative diseases is to reduce the inflammatory aspects of some neurodegenerative conditions [203]. Immune checkpoint inhibitors may potentially harm healthy cells unintentionally [204]. This is because they have the ability to interfere with ‘immunological checkpoint molecules’ interactions with the ligands on healthy cells [205]. ICIs are drugs that allow the body’s defenses to recognize and eliminate cells that are cancerous [206]. ICIs, which have been created for dealing with late-stage cancer, have lately grown in significance in oncology [206, 207]. Furthermore, several research found that these therapies are successful in easing and even correcting cognitive deficits as well as lowering disease pathology [208]. On the other hand, this can lead to a wide range of immune-related adverse events [209].

### The neurological immune-related adverse effects

**Fig. 4:**
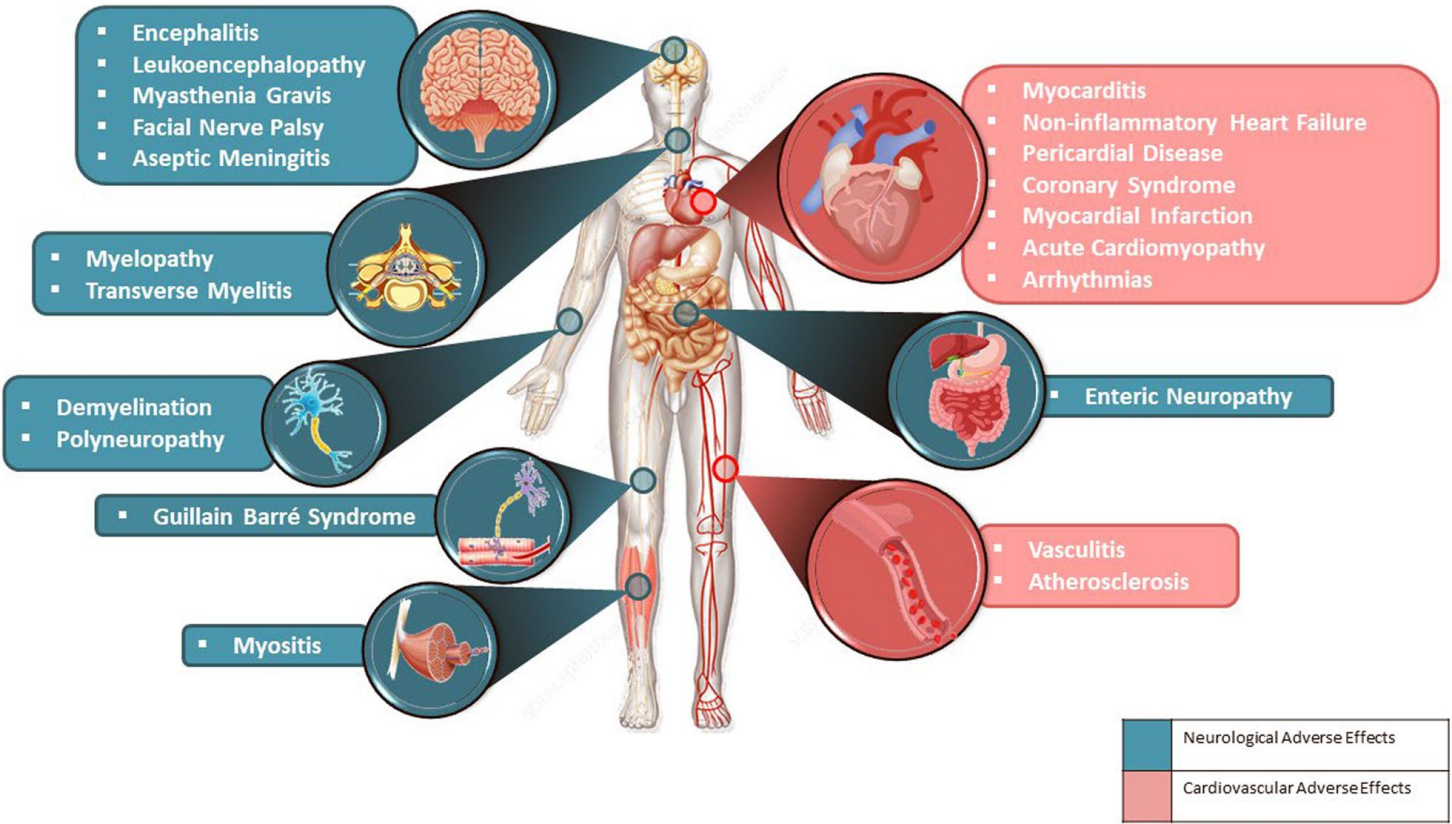
Adverse effects of immune checkpoint inhibitors in age-related neurodegenerative disorders

Encephalitis is one of the many immunological-related side effects that ICIs can trigger. The patient first had quickly been developing disorientation, a decreased state of awareness, headache, seizures, and localized neurological abnormalities. So, in patients receiving ICIs who report quickly developing confusion, encephalitis should be suspected [209]. Guillain–Barré syndrome, leukoencephalopathy, polyneuropathy, myelopathy, myasthenia gravis (MG), demyelination, facial nerve palsy, enteric neuropathy, enteric neuropathy, aseptic meningitis, transverse myelitis, and myositis are among the neurological problems caused by ICIs as shown in Fig. 4 [210, 211]. Symptoms of MG which occurred after ICI therapy had not been as “benign” as typical MG and, without a doubt, need recognition and concern with a greater degree of severity. Immune-related MG (irMG) is an uncommon but potentially lethal consequence. Anaerobic pneumonia and hypercapnic respiratory failure, which causes paralysis of the bulbar, were among the irMG-related fatalities. Diaphragmatic paralysis is the primary reason for mortality with irMG. Although irMG is uncommon, with the increased usage of ICIs, the number of people with irMG is expected to skyrocket [211].

### The cardiovascular immune-related adverse effects

Immune-related myocarditis is a potentially lethal non-neurological immune-related adverse event. A recent study indicated that diplopia and/or ptosis were symptoms of irMG and/or irMyopathy impacting ocular muscles, as well as asymptomatic myocarditis. Elevated levels of desmin and troponin (muscle-specific antigens) were found in both patients tumors, indicating the notion that targeting of skeletal and cardiac muscle stems from specific antigens found in the skeletal muscle, myocardium, and tumors that identical T-cell clones recognize them. As a result, ICIs patients with myositis of the ocular muscles should trigger further investigation of other muscles, especially the heart muscle [212]. The effects of ICIs on cardiovascular are more than just myocarditis. It is probably the most devastating immune-related cardiovascular complication, although there have also been reports of conduction problems, vasculitis, non-inflammatory heart failure, and pericardial disease. Freshly, data suggest that ICIs play a role in speeding atherosclerosis and boosting plaque inflammation, ultimately resulting in myocardial infarction as shown in Fig. 4 [213].

### Pre-existing autoimmune disease as a risk factor on cardiovascular safety

Myocarditis, arrhythmias, coronary syndromes, vasculitis, pericarditis, acute cardiomyopathy, and conduction abnormalities have all been documented as cardiovascular events (CVEs) [214–216]. With the rising use of ICIs, there has been significant concern about the health effects of ICI therapy in patients who have pre-existing autoimmune diseases, because these individuals are prone to aberrant anti-self-immune reactions [217, 218]. As a result, safety is restricted. Before beginning ICI therapy, patients should have an inclusive cardiovascular history taken, as well as a history of autoimmune illness [219].

### Potential strategies to overcome the limitations of immune checkpoint blockade

Although immunotherapy using ICIs is definitely one of the most promising techniques for the treatment of cancer and neurological illnesses, significant hurdles remain (e.g., low response rate, acquired resistance, and adverse events). A minority of individuals develop primary resistance to early immunotherapy, but the vast majority develop acquired resistance to ICIs [220]. Combination therapy, targeting alternative immune checkpoints, translational research, and biomarker identification are some of the potential options being investigated to overcome these constraints [220–222]. Combination therapy with different medications can help overcome immune checkpoint inhibitor resistance. Combining immune checkpoint inhibitors with other immunotherapies or chemotherapy, can increase response rates and overall survival [221]. Even so, the mechanisms underlying the development of ICB drug resistance are unclear [222]. Recently, several studies suggested that combining ICIs with anti-angiogenic drugs might be a potential treatment approach for overcoming the low efficacy of ICIs [220]. Targeting alternative immune checkpoints is a promising strategy that many studies supports. Alternative immune checkpoints such as T cell immunoglobulin and mucin-domain containing-3 (TIM-3), cytotoxic T lymphocyte-associated protein 4 (CTLA-4), and ligand lymphocyte activation gene-3 (LAG-3) can assist in overcoming PD-1 resistance [223]. In addition, there are many other immune checkpoints that could be targeted by ICB therapy. For example, targeting the PD-L2 checkpoint has shown promise in clinical trials [224]. Furthermore, translational studies and biomarker identification can help in decreasing the immune-related adverse events (irAEs) rate. Identifying biomarkers which predict response to ICIs can help select patients who will benefit the most from therapy. This can assist in eliminating wasteful therapy in patients who are unlikely to react, allowing for more personalized treatment [220, 225].

### Neurological immune-related AEs to manage

Because of the specific method of action of ICIs, they exhibit their own set of irAEs, as mentioned previously. Although neurologic immune-related AEs are infrequent, they are an arising area of concern due to the intricacies of the nervous system and the likelihood of long-term morbidity [214, 226]. Polyneuropathy, demyelination, 7th nerve palsy, MG, postinfectious polyneuritis, posterior reversible leukoencephalopathy, enteric neuropathy, transverse myelitis, encephalitis, and viral meningitis are among the neurologic occurrences recorded. It is crucial to exclude underlying malignant tumor progression, activity related to seizures, metabolic imbalance, and infection as potential reasons for neurological dysfunction [210]. There is a low threshold for withholding ICI medication in situations where symptoms are indicative of probable neurological damage, even if symptoms are of 'Grade 1' (mild) intensity. Corticosteroid medication should be started right away in patients with overt, progressing symptoms that have a functional impact (whether moderate or severe), and inpatient hospitalization should be considered. If there is any involvement of the respiratory muscles, patients should be admitted to centers where ventilation may be easily supported. An approach to investigation and management is summarized in Table 5 [227].

**Table 5:** An approach to the investigation and management of neurologic immune-related adverse events

Symptom grade	Diagnostic investigations	Management
Grade 1 (mild): no interference with function	History and neurological examination U&E, FBC, HbA1c, LFTs, TFTs, MMA, B12/Folate, HIV, homocysteine, and Autoimmune screen	Low threshold to hold ICPi and monitoring symptoms for progression
Grade 2 (moderate): some interference with ADLs NB: any cranial nerve involvement	*Suspected Central disorder* - MRI Head ± spine (T1, T2, STIR ± GAD) - Lumber Puncture- CSF + OCB and Viral PCR - Antibodies (NMDAR, LGi1, CASPR2, MOG, AQP4 and paraneoplastic)	- Withhold ICPi - Initial observation OR 0.5-1 mg/kg Prednisolone - Specialist neurology input - Empiric antibacterial and antivirals (encephalitis/meningitis)
	*Suspected peripheral disorder* - CK, ganglioside ab, AChR/MuSK ab, myositis ab panel, ± vasculitis screen - NCS, EMG (± SFEMG, RNS)	- Withhold ICPi - Initial observation OR 0.5-1 mg/kg Prednisolone - Specialist neurology input
Grade 3/4 (severe): limits self-care and aids warranted Respiratory symptomatology, e.g., dyspnea	*Suspected central disorder* - MRI head - Lumbar puncture- CSF + OCB and Viral PCR (suspicion of meningitis/ encephalitis) - Antibodies as above	- Admit patient - Permanently discontinue ICPi - IV methylprednisolone 2 mg/kg - Specialist neurology input - Empiric antibacterial and antivirals (CSF lymphocytosis or pyrexia
	*Suspected peripheral disorder* - Neurophysiology (RNS, SFEMG, EMG, NCS) - CK, ganglioside ab, AChR/MuSKab, myositis ab panel, ± vasculitis screen - Spirometry: FVC, FEV1 lying and standing (if neuromuscular respiratory involvement) - ECG/ cardiac monitoring if myositis or GBS presentation - Consider nerve/ muscle biopsy	- Admit patient and consider need for ICU admission - Permanently discontinue ICPi - IV methylprednisolone 2 mg/kg - Specialist neurology input - IVIG/ plasmapheresis

## Discussion

Neurodegenerative disorders encompass a variety of progressive diseases marked by the slow degeneration of nerve cells in either the brain or the peripheral nervous system. These illnesses, such as Alzheimer's disease and Parkinson's disease, cause a range of cognitive and motor deficits that worsen over time [228]. Alzheimer's disease is the predominant kind, characterized by memory loss, cognitive decline, and changes in behavior. Millions of individuals worldwide are affected by it, and it frequently results in death [229]. Meanwhile, Parkinson's disease primarily impacts motor function, leading to tremors, stiffness, and impaired balance. While not as common as Alzheimer's, Parkinson's disease also has a substantial impact on life expectancy. Both diseases pose difficulties for the healthcare sector due to their chronic nature and significant impact on patients quality of life [230].

Stem cell therapy, gene therapy, and the administration of medicines like metformin and rapamycin showed promise in treating neurodegenerative diseases. For example, stem cell therapy can decrease the accumulation of amyloid-beta, enhance cognitive function, and control motor symptoms in Alzheimer's disease by the transplantation of dopaminergic precursors [231]. However, the area faces challenges including the need for neurosurgical treatments, potential dangers associated with immunosuppression, and reported cases of tumor growth in both human and rodent studies [232–234]. In order to address these problems, further investigation is required to control the proliferation and specialization of stem cells, improve precision in targeting by utilizing molecular markers, develop effective delivery techniques, and allow the genetic and clinical diversities observed in individuals with Alzheimer's disease. While preclinical findings are encouraging, clinical trials are currently in their initial stages. Therefore, it is imperative to establish standard protocols and carry out a thorough examination to determine the safety, effectiveness, and enduring impacts of these treatments for neurodegenerative diseases.

Furthermore, notable advancements have been achieved in the management of neurodegenerative disorders, such as Alzheimer's disease, with the creation of specific treatments like aducanumab and lecanemab. Each target specifically targets the fundamental disease pathways that have the potential to produce significant clinical outcomes but may also result in distinct side effects identified during studies.

Aducanumab, approved by the FDA in 2021, targets amyloid-beta plaques, a pivotal factor in Alzheimer's disease progression. According to preclinical research and clinical trials, aducanumab may improve neurological function by lowering Aß plaques and increasing neuronal calcium permeability [235]. The ENGAGE and EMERGE trials demonstrated significant reductions in amyloid plaques, underscoring aducanumab's therapeutic potential. Aducanumab has also been linked to several adverse effects. Headache, diarrhea, and other constitutional symptoms are the most often reported. ARIA-E is among the most severe adverse effects seen in anti-Aβ monoclonal antibody therapy, including aducanumab [236]. Avgerinos et al. conducted a meta-analysis which revealed that aducanumab had the highest chance of developing ARIA-E when compared to other anti-Aβ antibody immunotherapies [237]. While deaths have been reported with other anti-Aβ immunotherapy treatments like bapineuzumab and solanezumab, no deaths have been associated with aducanumab in Alzheimer's disease treatment [238–240].

On the other hand, disparities in post hoc analysis have sparked doubt in the scientific and medical communities. Some studies indicate that while aducanumab's effects show statistical significance, their clinical relevance remains uncertain [241–243]. Critics argue that the FDA's initial denial of aducanumab approval may have been overly cautious and not fully considered other relevant aspects [244]. Confounding variables may have contributed to differences observed between the EMERGE and ENGAGE trials, emphasizing the need for additional studies to bolster confidence in aducanumab's efficacy [241]. Despite these concerns, aducanumab consistently demonstrated significant reductions in amyloid across all examined cortical brain regions [236, 245].

On the other hand, lecanemab specifically targets tau protein aggregates, which are also a characteristic feature of Alzheimer's disease. Further trials are presently assessing its efficacy and safety. The primary adverse effects are infusion-related reactions, which are typical with monoclonal antibody therapies [246]. These medications show targeted approaches to treating AD, emphasizing the importance of precisely balancing therapeutic efficacy with safety considerations during the development of treatment options for Alzheimer's disease.

Developing immunotherapy to target α-Syn aggregates in synucleinopathies like Parkinson's disease is a multifaceted challenge that demands a comprehensive strategy rooted in the latest scientific insights. Preclinical trials investigating treatments aimed at reducing α-Syn aggregates and improving motor and cognitive abilities in experimental animals have shown promising results [171, 180–182]. These findings suggest potential therapeutic benefits for α-Syn targeting in neurodegenerative diseases.

However, the translation of these promising preclinical outcomes to clinical trials has proven complex and variable. Clinical studies evaluating immunotherapeutic drugs directed at α-Syn have produced mixed results. For instance, BIIB054/cinpanemab, which targets the N-terminus of α-Syn, did not meet expectations in a pivotal phase II trial [188]. On the other hand, PRX002/prasinezumab, which targets the C-terminus of α-Syn, showed favorable results in secondary endpoints, prompting the commencement of a phase IIB trial [193, 247].

These discrepancies highlight the challenges and uncertainties in moving from preclinical success to clinical efficacy in treating neurodegenerative diseases. Issues such as target specificity, dosing protocols, and patient heterogeneity can significantly influence trial outcomes. Therefore, further research and larger-scale studies are essential to elucidate the potential of α-Syn-targeting immunotherapies in clinical practice.

Moreover, recent findings from clinical trials, such as NCT03318523 (clinicaltrials.gov/ct2/show/NCT03318523), underscore the difficulty in translating biomarker discoveries into meaningful clinical impacts. While these trials enrolled early Parkinson's disease patients meeting biomarker criteria, they did not establish clear links between disease progression and treatment effects on biomarker measurements like those of cinpanemab. This underscores the ongoing need for reliable biomarkers that accurately gauge early PD progression. Such biomarkers could not only accelerate the development of tailored therapies, but also enhance the efficacy of immunotherapy strategies for managing synucleinopathies.

Both aducanumab and α-Syn-targeting immunotherapy are advanced approaches in the treatment of neurodegenerative diseases. Continued research endeavors are essential to gain a deeper understanding of their mechanisms, refine therapeutic treatments, and validate their clinical advantages. Realizing the full therapeutic promise of these novel techniques in treating Alzheimer's disease and synucleinopathies such as Parkinson's disease will require advancing our understanding of disease biology, enhancing treatment specificity, and discovering potent biomarkers.

## Conclusions

Recent studies show that various immunotherapies, both active and passive, can significantly improve outcomes in AD and PD. Aducanumab and lecanemab, targeting Aβ and tau aggregates in AD, respectively, are notable advancements. Aducanumab, FDA-approved in 2021, reduces Aβ plaques but has side effects like ARIA-E. Research is ongoing to validate its clinical relevance despite mixed trial results.

Immunotherapy for PD targeting α-syn shows promise but faces challenges such as crossing the BBB and achieving clinical efficacy. Biomarkers are key for tracking disease and treatment progress, though most research is in animal models. The human applicability of α-syn processes remains unclear. Some reviewed studies did not demonstrate the efficacy of α-syn antibodies in PD, but future technological advancements may address these issues.

## Data Availability

No data associated in the manuscript.

## Abbreviations

AD: Alzheimer's disease
PD: Parkinson's disease
MSCs: Mesenchymal stem cells
Aβ: Amyloid-beta-peptide
AD-MSCs: Adipose tissue-derived mesenchymal stem cells
PDD: Parkinson's disease dementia
NMDA: *N*-Methyl-d-aspartate
AAV: Adeno-associated virus
PD-1: Programmed cell death protein 1
APCs: Antigen-presenting cells
ICIs: Immune checkpoint inhibitors
TCR: T cell receptor
EAE: Experimental autoimmune encephalomyelitis
MHC: Major histocompatibility complex
Th1: T helper cell 1
SCI: Spinal cord injury
FDA: Food and Drug Administration
ARIA-E: Amyloid-related imaging abnormalities-edema
ARIA-H: Amyloid-related imaging abnormalities-cerebral microhemorrhage
IgG1: Immunoglobulin 1
MCI: Mild cognitive impairment
ICB: Immune checkpoint blockade
MG: Myasthenia gravis
IrMG: Immune-related MG
IrMyopathy: Immune-related myopathy
CVEs: Cardiovascular events
CTLA-4: Cytotoxic T lymphocyte-associated protein 4
TIM-2: T cell immunoglobulin and mucin-domain containing-3
LAG-3: Ligand lymphocyte activation gene-3
PD-L2: Programmed cell death-ligand 2
IrAEs: Immune-related adverse events
AChR: Acetylcholine receptor
HbA1c: Glycated hemoglobin
ICI: Immune checkpoint inhibitor
